# Incidental rewarding cues influence economic decisions in people with obesity

**DOI:** 10.3389/fnbeh.2015.00278

**Published:** 2015-10-15

**Authors:** Jakob Simmank, Carsten Murawski, Stefan Bode, Annette Horstmann

**Affiliations:** ^1^Junior Research Group ‘Decision-making in obesity’, IFB Adiposity Diseases, Leipzig University Medical CenterLeipzig, Germany; ^2^Department of Neurology, Max Planck Institute for Human Cognitive and Brain SciencesLeipzig, Germany; ^3^Department of Finance, The University of MelbourneMelbourne, Victoria, Australia; ^4^Decision Neuroscience Laboratory, Melbourne School of Psychological Sciences, The University of MelbourneVictoria, Australia; ^5^Collaborative Research Centre, Leipzig University Medical CenterLeipzig, Germany

**Keywords:** inter-temporal decision-making, obesity, priming, delay discounting, gender, eating behavior, decision-making

## Abstract

Recent research suggests that obesity is linked to prominent alterations in learning and decision-making. This general difference may also underlie the preference for immediately consumable, highly palatable but unhealthy and high-calorie foods. Such poor food-related inter-temporal decision-making can explain weight gain; however, it is not yet clear whether this deficit can be generalized to other domains of inter-temporal decision-making, for example financial decisions. Further, little is known about the stability of decision-making behavior in obesity, especially in the presence of rewarding cues. To answer these questions, obese and lean participants (*n* = 52) completed two sessions of a novel priming paradigm including a computerized monetary delay discounting task. In the first session, general differences between groups in financial delay discounting were measured. In the second session, we tested the general stability of discount rates. Additionally, participants were primed by affective visual cues of different contextual categories before making financial decisions. We found that the obese group showed stronger discounting of future monetary rewards than the lean group, but groups did not differ in their general stability between sessions nor in their sensitivity toward changes in reward magnitude. In the obese group, a fast decrease of subjective value over time was directly related to a higher tendency for opportunistic eating. Obese in contrast to lean people were primed by the affective cues, showing a sex-specific pattern of priming direction. Our findings demonstrate that environments rich of cues, aiming at inducing unhealthy consumer decisions, can be highly detrimental for obese people. It also underscores that obesity is not merely a medical condition but has a strong cognitive component, meaning that current dietary and medical treatment strategies may fall too short.

## Introduction

Obesity is associated with a positive energy balance: Energy intake exceeds energy expenditure. In this context, dietary choices seem to be a crucial factor to the development and maintenance of obesity. Dietary choices can be characterized by the trade-off between highly palatable, high-calorie and easily accessible but often less healthy (e.g., convenience or take-away) food, and more healthy food contributing to the long-term maintenance of normal-weight. Paradoxically, subjects with obesity often exhibit a preference for high-calorie food despite having dietary goals to the contrary.

How can this behavior be explained? First, obesity may be paralleled by a general preference for immediately consumable rewards, i.e., highly palatable, high-calorie food. Second, choice behavior might be less stable in general and thus might often produce decisions that are not in line with subjective dietary goals. Third, choice behavior of subjects with obesity may be easily disturbed by internal or external factors such as stress or incidental rewarding cues in the environment. Differences between lean and obese people in the stability of inter-temporal decision-making preferences to external cues would have serious implications for our understanding of obesity. The abundance of food-related cues in our everyday life requires a certain robustness of decision-making preferences in order to maintain normal weight. Hence, instability in decision-making preferences is likely to be detrimental, because it might lead to the neglect of long-term dietary and weight maintenance goals and consequently to obesity.

Recent research indeed suggests obesity-associated differences in reward-based and impulsive decision-making even outside the food context. For example, obese subjects exhibited difficulties in inhibiting prepotent responses in a stop-signal task (Nederkoorn et al., 2006) and in a Go/No-Go task (Batterink et al., 2010; Kamijo et al., 2012), and chose immediate rewards at the expense of higher future losses more often than controls in the Iowa Gambling Task (Pignatti et al., 2006; Brogan et al., 2010; Horstmann et al., 2011). In animal models it has been shown that intertemporal decision-making preferences might depend on diet, the amount of body fat mass, or the hormonal regulation of energy homeostasis. Leptin-deficient obese rats more often did not wait for a delayed but bigger portion of food (Boomhower et al., 2013) and exhibited an overall higher degree of sensitivity to reinforcement (Buckley and Rasmussen, 2012). Moreover, the behavior of rats that have been fed by a high-fat diet was more sensitive to antidopaminergic agents than the behavior of rats fed by a standard-chow diet. After administering Haloperidol they showed a more pronounced increase in future discounting (Boomhower and Rasmussen, 2014). This suggests that the interplay of diet and dopaminergic transmission influences intertemporal decision-making. In contrast, human studies directly investigating inter-temporal decision-making using monetary rewards have produced ambiguous results so far. Inter-temporal decision-making, i.e., deciding between immediate rewards, which are smaller in size, and rewards, which are delayed in time but have an overall higher value, essentially mirrors the trade-off in dietary choice described above. Studies investigating inter-temporal decision-making using delay discounting tasks in obesity showed either no differences between lean and obese subjects (Nederkoorn et al., 2006), behavioral differences were accounted for by differences in socioeconomic status (Davis et al., 2010), or differences were observed in obese women only (Weller et al., 2008). Thus, it is still unclear whether obese people's preference for immediate rewards in the food context can be transferred to the context of inter-temporal decision-making with monetary rewards.

Further, little is known about the overall stability of decision preferences and their susceptibility to environmental cues in obese compared to lean people. However, research on eating behavior suggests a highly consistent positive relationship between body mass index (BMI) and the “disinhibition” subscale of the Three Factor Eating Questionnaire (TFEQ; Stunkard and Messick, 1985), also conceptualized as “opportunistic eating' (Bryant et al., 2008; Hays and Roberts, 2008; Dietrich et al., 2014; for a review on the impact of different eating behavior scores on BMI see: French et al., 2012). Opportunistic eating describes the lack of control over eating, especially in the presence of tempting external cues or situations. Recently, it has been shown experimentally that obese men respond habitually with appetitive behavior to cues signaling the availability of food reward even in the absence of subjective food motivation (Horstmann et al., 2015a). In support, the rise in the prevalence of obesity coincided with a significant change in food environment (Hill and Peters, 1998; Leung et al., 2011) with highly palatable and often unhealthy food being available virtually everywhere (“obesogenic environment”; cf. Jeffery and Utter, 2003). Consistently, previous research suggests that obese subjects' preferences in the context of eating behavior are susceptible to external cues, which are incidental to the decision at hand, e.g., a higher degree of “opportunistic eating” (Bryant et al., 2008; Hays and Roberts, 2008), signifying instability toward environmental cues, the emotional state present at the moment of decision (“emotional disinhibition,” Hays and Roberts, 2008), induced stress (for a review see: Scott and Johnstone, 2012), and a stronger responsiveness to food in general (Carnell and Wardle, 2007, 2008; García-García et al., 2014). Moreover, opportunistic eating entails a set of personality characteristics that likely ranges beyond eating behavior (cf. Bryant et al., 2008). This raises the important question whether the susceptibility to external cues that obese subjects exhibit in the food context can be generalized to other domains of decision-making.

The present study addresses three important open questions: Firstly, we address a potential difference in inter-temporal preferences between lean and obese people. Further, we investigate the general stability of delay discounting task performance and the stochasticity of decisions in lean and obese subjects. Thirdly, we investigate differences in the susceptibility to priming between lean and obese participants by investigating the contextual stability of decision-making behavior toward external cues in both groups. In order to test for the stability of discounting task performance, the same group of participants underwent two delay discounting task sessions. Additionally, in order to test for the susceptibility of reward-based decision-making toward incidental cues, participants completed a behavioral priming task. We administered a standard delay discounting task which entailed an additional priming session in which participants where presented with rewarding pictures of different context categories. Behavioral priming has been proven to be effective in a number of circumstances (Strahan et al., 2002; Dijksterhuis et al., 2005; Guitart-Masip et al., 2010; Bijleveld et al., 2012), including delay discounting tasks (Van den Bergh et al., 2008; Murawski et al., 2012; Kim and Zauberman, 2013; Van der Wal et al., 2013; Luo et al., 2014).

## Methods

### Subjects

Fifty-two subjects (26 female) were recruited from the participant database of the Max Planck Institute for Human Cognitive and Brain Sciences in Leipzig, Germany. They were screened with regard to inclusion and exclusion criteria during an initial telephone interview. Inclusion criteria were (i) Body Mass Index (BMI) either between 18.5 and 25 kg/m^2^ (lean group) or 30 and 40 kg/m^2^ (obese group), (ii) age between 18 and 35 years, and (iii) normal or corrected-to-normal vision (for demographics see Table 1). Exclusion criteria were (i) any medical condition, except for hypertension, (ii) current medication, except for anti-hypertensives and oral contraceptives, (iii) current or past diagnosis of an addictive disorder, including smoking, (iv) a history of mood disorders, eating disorders or neuropsychological disorders, including anxiety disorders and obsessive compulsive disorders (OCD).

**Table 1:** **Participant demographics**.

	**Lean**	**Obese**	***t***	***p***	**Male**	**Female**	***t***	***p***
BMI	22.6±1.52	34.69±2.61	20.41	0.01	28.78±6.18	28.51±6.85	0.15	0.88
Age	25.96±3.33	27.08±4.2	1.06	0.3	26.88±3.27	26.15±4.3	0.69	0.49
WMT score	16.38±4.26	17.27±4.56	0.72	0.47	17.42±3.64	16.23±5.04	0.98	0.33
	**Lean**	**Obese**	**Mann-Whitney-U**	***p***	**Male**	**Female**	**Mann-Whitney-U**	***P***
Household income	1.5±0.71	2.03±0.82	216	0.02	1.77±0.76	1.77±0.86	332	0.91
Parents' household income	1.73±0.83	1.81±0.63	297.5	0.55	1.65±0.69	1.88±0.77	282	0.27
Contentment with current income	3.0±0.87	2.81±0.82	297.5	0.43	2.81±0.75	2.96±0.82	308	0.56
Secondary school education	2.81±0.4	2.65±0.63	307	0.44	2.81±0.49	2.65±0.56	288	0.21
Professional qualification	3.7±1.26	3.31±1.32	284	0.3	3.73±1.12	3.27±1.43	282	0.28

Demographics by gender and weight status groups (mean ± s.d.). BMI = body mass index = bodyweight/height^2^ in kg/m^2^; WMT score = Values from Wiener Matrizzen Test, Formann and Piswanger (1979); Household income = 3-point scale ranging from low (0–700€) over intermediate (701–1300€) to high (>1300€); Parent's annual household income = 3-point scale ranging from low (< 30000€ per annum) over intermediate (30000–60000€ per annum) to high (>60000€ per annum); Contentment = 4-point scale ranging from “much too little” over “too little” and “sufficient” to “I don't have to worry about money”; Secondary school education = 3-point scale from “no secondary school qualification” to “A level”; Professional qualification = 5-point scale ranging from “no qualification” to “master's degree.”

Participants' height and weight were measured in their first laboratory session to confirm self-reported values. Further, participants completed the Beck Depression Inventory (BDI, Beck et al., 1996) and the Yale Food Addiction Scale (YFAS, Gearhardt et al., 2009), and were excluded from the study when exceeding the cut-off value of 18 on the BDI or meeting the criteria for “Food Addiction” on the YFAS.

The study was carried out in accordance with the Declaration of Helsinki and was approved by the local ethics committee of the University of Leipzig. Participants gave their written informed consent before taking part in the study. They were reimbursed with a base payment of €7 per hour. In addition, they had a chance to win a monetary reward that depended on their choices in the experimental session (see below).

### Questionnaires

To control for the most important factors that potentially affect inter-temporal decision-making, we measured general intelligence (cf. Shamosh and Gray, 2008), education level, and household income (cf. Green et al., 1996). General intelligence was assessed by an adapted version of Raven's progressive matrices, the Wiener Matrizen-Test (Formann and Piswanger, 1979), while secondary school qualification, professional qualification, and household income were assessed by a short education and income questionnaire (see Table 1).

Other potential confounding factors were impulsivity and sensitivity to reward (SR; De Wit et al., 2007). We assessed impulsivity using the U-P-P-S impulsivity questionnaire (Whiteside and Lynam, 2001) and the 15-item German version of the Barratt-Impulsiveness Scale 11 (Patton et al., 1995; Meule et al., 2011), and SR using the Behavioral Inhibition System and Behavioral Activation System questionnaire (BIS/BAS, Carver and White, 1994).

Delay discounting has been linked to subjective time perception (Zauberman et al., 2009; Han and Takahashi, 2012; Cooper et al., 2013). Thus, we additionally assessed participants' subjective perception of the length of objective time horizons (Zauberman et al., 2009; Cooper et al., 2013).

### Inter-temporal decision-making

Each participant completed two experimental sessions that took place on different days (mean number of days between sessions 63.1; SEM = 5.3). In the first session (Baseline session) differences in inter-temporal decision-making between obese and lean subjects were assessed using a standard delay-discounting task. In the second session (Priming session), temporal stability of inter-temporal preferences was assessed as well as the influence of incidental reward cues on inter-temporal decision-making.

#### Delay-discounting task

Participants completed a computerized version of a delay-discounting task, implemented in Matlab and the Psychophysics Toolbox. In each trial, participants were asked to choose between a monetary reward that was available immediately but smaller (smaller and sooner option, SS), and a monetary reward that was larger but only available after a delay (larger and later option, LL); for example, €20 today vs. €32.50 in 4 months. Participants were instructed to choose the option that they prefer and to evaluate each decision independently of all other decisions.

##### Trial procedure

Each trial started with a screen on which the two choice options, i.e., the two amounts of money together with their respective delays (SS and LL options), were presented for 3 s, one above a fixation cross at the center of the screen and one below the fixation cross. Subsequently, the same choice options were displayed in a horizontal arrangement, initiating the response stage (2 s) in which participants had to indicate their choice via a key press. Positions of SS and LL options on the screen (top/bottom and left/right) were counterbalanced across the task. Initially, 10 practice trials were presented to familiarize participants with the task procedure. During these trials the experimenter was present to answer questions and ensure that the participant understood the instructions.

##### Incentive compatibility of rewards

To incentivise participants to reveal their preferences, we rendered the rewards in the task incentive-compatible: At the end of each session, each participant had a 1-in-6 chance (dice roll) to win one of the choices made during the session. If the participant won, a trial was chosen at random and the participant was paid the chosen amount at the chosen delay. The money was transferred to the participants' bank account after the respective delay.

#### Baseline session

The aim of the first experimental session was to estimate a participant's discount function. To obtain precise estimates we adopted a two-step procedure. In the first step, a titration task (“Dynamic Adjustment task,” DA) was administered to obtain a first set of (approximate) parameter estimates. In the second step, a randomized choice task (“Random Choice task,” RC), parameterized based on the DA task, was used to validate the parameters estimated in the DA task and to improve the precision of parameter estimates.

##### Dynamic adjustment task

Discount rates vary widely across the population. The DA task approximated each participant's indifference points (*ip*) by dynamically adjusting the percentage difference (*r*) between SS (always immediate) and LL amounts using a staircase procedure. The task terminated when the difference between the previous and the newly computed value of *r* was sufficiently small (< 0.015). We determined *ip*s for six delays (1/2/4/6/9/12 months). Due to the adaptive nature of this task, trial numbers differed between subjects.

##### Random choice task

In the Random Choice (RC) task participants were presented with a series of choices between SS and LL rewards, which were calibrated based on the results of the DA task. At each of six delays (1/2/4/6/9/12 months), six different amounts were shown. The amounts were multiples of the *ip* obtained in the DA task for the particular delay [0.25; 0.55; 0.85; 1.15; 1.45; 1.75]. Each amount x delay combination was administered 4 times, resulting in 144 trials (6 amounts/delay × 6 delays × 4 repetitions). Amount/delay combinations were presented in random order.

We recorded reaction times (RT) in both tasks.

#### Priming session

In the second session participants completed the DA task followed by a RC task. In this session, only two delays (2 and 4 months) were used. The two tasks (DA and RC) provided precise estimates of participants' discount function parameters and validated them, as described above. Subsequently, participants completed a Primed Random Choice task (PRC). In the PRC task, participants' susceptibility to incidental external cues, which were hypothesized to systematically affect inter-temporal decision-making, was assessed. The cues used in the task were rewarding pictures from different context categories. The first category of images contained pictures depicting couples engaging in sexual behavior (erotic condition). Prior research showed that erotic pictures bias a person's choice behavior toward more present-oriented choices in a delay-discounting task (Van den Bergh et al., 2008; Kim and Zauberman, 2013). The second category contained images of highly palatable food items (food condition). Given the fact that obese people perceive and process food cues differently than lean people (e.g., Rothemund et al., 2007; Wang et al., 2009; Nijs et al., 2010), we expected a differential effect of these pictures depending on weight status groups. The third and last category contained images of happy old people and family scenes (social condition), which can be conceptualized—in contrast to pictures from the first category—as secondary reinforcers. However, as obese subjects are highly stigmatized by society (for a review: Puhl and Heuer, 2009), it is likely that they perceived the pictures, similar to the pictures from the food condition, differently than lean.

##### Priming image selection

All images used in the PRC task were selected from the International Affective Picture System (IAPS; Lang et al., 1997; see Supplementary Material for numbers). All pictures were of high positive valence in order to be perceived as generally rewarding (see norm ratings in Lang et al., 2008). Images were selected in two steps. In the first step, we identified a set of candidate images that fitted into one of the three context categories described above. In the second step, the candidate images were rated by an independent sample of 44 participants with demographic characteristics comparable to the study sample [23 lean (12 female), 21 obese (11 female); mean age (obese) = 27.52 (SD = 3.66), mean age (lean) = 25.83 (SD = 3.07); mean BMI (obese) = 34.32 (SD = 2.31), mean BMI (lean) = 22.33 (SD = 1.62); *t*-test for group difference showed no significant difference for age: *t*_(43)_ = 0.68; *p* > 0.05, but a significant group difference for BMI: *t*_(43)_ = 20.31; *p* < 0.01]. Ratings of perceived valence and arousal of the images were assessed using a nine-point rating scale.

For each of the three context categories described above, we selected six images such that all 18 images had high positive valence and high arousal, and that only two pictures exhibited statistically significant group differences in the mean ratings of valence or arousal [arousal rating of IAPS picture 4695 (erotic condition): men M (SD) = 1.76 (0.94), women M (SD) = 2.87 (2.26), *t*_(42)_ = 2.15, *p* = 0.03; valence rating of IAPS picture 7405 (food condition): lean M (SD) = 2.43 (1.85), obese M (SD) = 3.76 (1.61), *t*_(42)_ = 2.52, *p* = 0.02; see Table 2 and Supplementary Material].

**Table 2:** **IAPS pictures used for priming**.

**Context category**	**IAPS number**	**Motif depicted**	**Valence**	**Arousal**
Erotic condition	4695	Naked couple	1.84 ± 1.41	6.53 ± 2.04
	4645	Kiss	1.68 ± 1.07	4.65 ± 2.43
	4650	Intimate naked couple	2.39 ± 1.30	5.84 ± 2.18
	4676	Naked couple	1.80 ± 1.32	6.55 ± 2.10
	4693	Naked couple	2.02 ± 1.41	5.57 ± 2.55
	4599	Intimate couple	1.89 ± 1.02	5.52 ± 2.09
Food condition	7351	Pizza	2.95 ± 2.00	3.89 ± 1.82
	7400	Chocolate	3.50 ± 1.86	3.73 ± 1.81
	7405	Muffins	3.27 ± 1.98	3.59 ± 1.83
	7470	Pancakes	3.09 ± 2.07	3.75 ± 1.93
	7480	Pasta	2.7 ± 1.8	3.20 ± 1.97
	7481	Casserole	2.45 ± 1.37	4.27 ± 2.2
Social condition	2152	Mother and infant	2.09 ± 1.96	3.32 ± 2.09
	2165	Father hugging child	1.93 ± 1.58	3.75 ± 2.29
	2370	Laughing old men	3.09 ± 2.03	3.30 ± 1.94
	2495	Old man	4.50 ± 1.84	3.25 ± 2.01
	2500	Old man	3.05 ± 1.70	3.18 ± 2.08
	2510	Laughing old lady	2.93 ± 2.25	2.95 ± 1.51

*Rating scores for priming images (mean ± s.d.). Valence ranging from 1 (= very high valence) to 9 (= very low valence) and Arousal ranging from 1 (= very low arousal) to 9 (= very high arousal)*.

##### Primed random choice task

The subsequent Primed Random Choice task (PRC) task assessed (i) whether incidental reward cues, as operationalized by the positive images, can bias participants' inter-temporal decision-making, and (ii) whether the factors weight status and gender are associated with the extent to which these images bias participants' behavior in inter-temporal decision-making.

**Figure 1:**
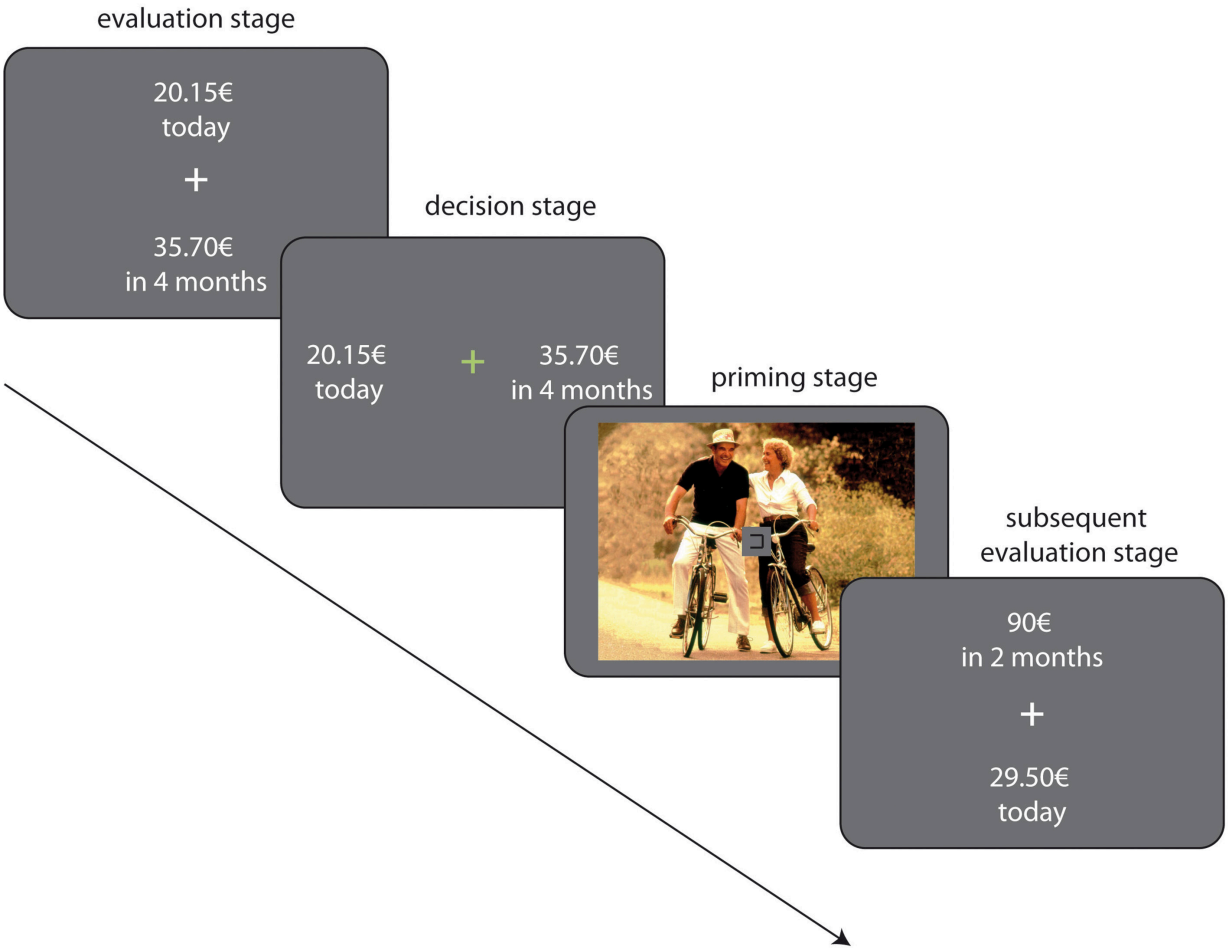
**Illustration of the experimental paradigm**.

Each trial in the task consisted of two stages, a priming stage and a decision stage. During the priming stage, one picture from the picture set was randomly drawn and presented for a jittered duration of 3–5 s (sized 375 × 500 pixels, displayed at the center of the screen). To withdraw attention from the images during the priming stage, we included a sustained attention task: simultaneously to picture onset, a box appeared at the center of the screen (see Figure 1). The left and right sides of the box opened and closed again in 500 ms intervals between 3 and 4 times in random order while the picture was displayed (Tusche et al., 2010). Participants had to indicate via key press which side of the box was open. Note, however, that the images were still clearly visible and could be visually processed. Immediately following image presentation the choice options (SS/LL rewards) were displayed, followed by a response screen (see RC task above). In each trial, one of 12 LL amounts was shown, which were multiples of the indifference point estimated in the preceding RC task (0.1; 0.15; 0.65; 0.75; 0.85; 0.95; 1.05; 1.15; 1.25; 1.35; 1.85; 1.9). The large number of amounts per delay allowed us to separately investigate easy and difficult trials. We defined difficult trials as trials in which the LL amount was close the participant's indifferent point at that given delay, i.e., close to *r* = 1 (see Table 3). We hypothesized that difficult decisions were the most likely to be affected by incidental rewarding cues whereas easy trials, i.e., trials in which the LL amount was far away from the indifference point, were less susceptible to influences by incidental cues. Amount/delay combinations were randomly presented, and each combination was presented three times in each of the following five conditions: (a) a neutral condition in which choices were not primed, that is, no picture was shown, (b) the erotic priming condition, (c) the food priming condition, (d) the social priming condition and, lastly, (e) a condition in which the presentation of a random picture was not followed by a delay discounting trial. All together the task session consisted of 288 trials. After completion of the PRC task, participants rated all images shown in the task using the same procedure as described for the Rating task (see above).

**Table 3:** **Reaction times**.

	**Session-task**	**Lean**	**Obese**	***F*_(1, 50)_**	***p***	**Male**	**Female**	***F*_(1, 50)_**	***p***
All trials	Baseline-DA	1088	1079	0.04	0.84	1128	1040	4.31	0.04
	Baseline-RC	1030	970	2.06	0.16	1060	940	9.18	0.00
	Baseline-sum	984	973	0.15	0.70	1009	948	6.00	0.02
	Priming-DA	1036	997	0.79	0.38	1058	974	3.78	0.06
	Priming-RC	1015	942	2.73	0.11	1020	937	3.68	0.06
	Priming-PRC	945	865	3.84	0.06	951	858	5.32	0.03
	Priming-sum	944	893	2.24	0.14	962	875	6.98	0.01
Easy trials	Baseline-RC	1005	947	1.74	0.19	1026	926	8.20	0.01
	Priming-RC	974	892	3.31	0.08	977	889	3.91	0.05
	Priming-PRC	912	825	4.97	0.03	923	814	8.25	0.01
	Priming-sum	923	837	5.13	0.03	933	827	8.20	0.01
Difficult trials	Baseline-RC	1065	998	2.17	0.15	1104	959	4.28	0.04
	Priming-RC	1050	993	1.44	0.24	991	906	1.50	0.23
	Priming-PRC	962	903	1.80	0.19	1051	993	4.40	0.04
	Priming-sum	978	919	1.95	0.17	978	887	4.28	0.04

*Reaction Times in milliseconds by gender and weight status groups. Difficult trials are trials in which the SS/LL combination is close to the participant's ip whereas in easy trials the SS/LL combination is further away from the ip. (EASY = for RC, r = 0.25 × ip and r = 1.75 × ip; for PRC, r = 0.1 × ip, r = 0.15, × ip, r = 1.85 × ip, r = 1.9 × ip; DIFFICULT = for RC, r = 0.85 × ip, r = 1.15 × ip; for PRC, r = 0.85 × ip, r = 0.95 × ip, r = 1.05 × ip, r = 1.15 × ip). One-way ANOVAs were used to assess group differences. Priming-sum, mean of all easy/difficult trials of the Priming session*.

#### Estimation of discount function

To model participants' choices, we assumed that participants value choice options according to a discount function. We further assumed that the probability of choosing a particular option in a given trial was given by the softmax function (see e.g., Kable and Glimcher, 2007). More precisely, we assumed that in each trial a participant chooses the larger, later amount (LL) with probability

$$p(LL)=\frac{1}{1+e^{-\frac{SV(LL)-SV(SS)}{s}}}$$

where *SV(SS)* and *SV(LL)* are the subjective values of the smaller, sooner and the larger, later amount, respectively, and *s* is the variance of the logistic distribution (*1/s* is often referred to as the gain of the softmax function).

To determine the shape of participants' discount function, we fitted three different candidate models to participants' choices and compared goodness-of-fit statistics of those models. In line with previous literature, we considered the exponential model (e.g., Samuelson, 1937), the hyperbolic model (e.g., Mazur, 1987), and the quasi-hyperbolic model (e.g., Laibson, 1997). In the exponential model, the discount function is given by

$$SV=\frac{1}{{\left(1+r\right)}^{\tau }},$$

where *r* denotes the discount rate and τ denotes the delay. The hyperbolic discount function is given by

$$SV=\frac{1}{1+k\tau },$$

where *k* denotes the discount rate and τ denotes the delay. Finally, in the quasi-hyperbolic model, the discount function is given by

$$SV=\beta \delta^{\tau },$$

where β denotes the present bias, δ denotes the discount factor and τ denotes the delay. In contrast to the discount factor δ describing the decrease of subjective value over time, the present bias β is a fixed discount of delayed rewards, irrespective of delay.

In addition to these parameters, we estimated *s*, the variance of the logistic distribution. The larger the parameter *s*, the shallower the softmax function and the lower the sensitivity of a participant's choices to differences in the values of the two choice options.

Parameters of the discount function and softmax model were estimated based on participants' choices in the RC task using maximum likelihood estimation (“fmin” function in Matlab).

## Results

### Questionnaires

Obese participants were more likely to have a high or intermediate household income than lean participants but showed no significant difference in education, parents' household income, contentment with current income, and IQ scores. No gender differences were found within or between groups for any of these variables (for all details and statistics see Table 1).

We found no significant differences between obese and lean participants on scores or sub-scores of the Barratt Impulsiveness Scale, the U-P-P-S impulsivity questionnaire and the BIS/BAS questionnaire. However, women compared to men (across weight groups) exhibited higher scores on the Behavioral Inhibition Scale and the U-P-P-S Urgency scale and lower scores on the U-P-P-S Sensation Seeking scale (Table 4). Testing for gender × weight status interactions revealed that women in the lean group had higher BIS scores than men in this group while this interaction proved only trend-significant in the obese group [lean women M (SD) = 20.31(3.88), lean men M (SD) = 17.38 (3.0), *t*_(24)_ = 2.15, *p* = 0.04; obese women M (SD) = 19.92 (3.73), obese men M (SD) = 17.08 (4.6), *t*_(24)_ = 1.74, *p* = 0.1]. Similarly, women in the lean group exhibited higher U-P-P-S Urgency scores than lean men while this interaction did not prove significant for the obese group [lean women M (SD) = 27.69 (6.0), lean men M (SD) = 23.15 (3.91), *t*_(24)_ = 2.29, *p* = 0.03; obese women M (SD) = 28.54 (5.8), obese men M (SD) = 24.92 (6.13), *t*_(24)_ = 1.55, *p* = 0.14]. No other significant interactions were found.

**Table 4:** **Impulsivity and Sensitivity to Reward/Punishment questionnaires**.

	**Lean**	**Obese**	***t***	***p***	**Male**	**Female**	***t***	***p***
U-P-P-S Urgency	25.42±5.47	26.73±6.13	−0.81	0.42	24.04±5.12	28.12±5.8	−2.69	0.01
U-P-P-S (Lack of) Perseverance	24.35±4.14	24.27±4.35	0.71	0.94	23.69±3.4	24.92±4.3	−1.15	0.26
U-P-P-S (Lack of) Premeditation	18.85±3.36	18.04±4.42	0.69	0.50	19.38±3.79	17.5±4.49	1.64	0.11
U-15-item version of the Barratt-impulsiveness Scale 11 (Patton et al., 1995; Meule et al., 2011)	34.27±6.44	32.38±6.79	1.03	0.31	35.15±5.09	31.5±7.52	2.05	0.05
BIS	18.85±3.71	18.5±4.35	−0.31	0.76	17.23±3.8	20.12±3.73	−2.76	0.01
BAS	40.73±3.61	41.85±4	−1.06	0.29	40.58±3.96	42±3.56	−1.63	0.18
BAS drive	12.38±1.96	12.46±2.01	−0.14	0.89	12.23±2	12.62±1.98	−0.70	0.49
BAS fun	12±1.52	12.46±1.56	−1.08	0.29	12.04±1.46	12.42±1.63	−0.90	0.37
BAS reward	16.35±1.52	16.92±1.81	−1.24	0.22	16.31±1.67	16.96±1.66	−1.42	0.16
BIS-15	30.19±4.32	30.42±5.62	−0.17	0.87	30.04±4.51	30.58±5.45	−0.39	0.70
BIS-15 non-planning	10.54±2.73	10.42±2.4	0.16	0.87	10.81±2.55	10.15±2.56	0.92	0.36
BIS-15 motor	10.88±2.34	11.27±2.71	−0.55	0.59	10.5±2.32	11.65±2.61	−1.69	0.10
BIS-15 attention	8.77±2.05	8.73±2.4	0.06	0.95	8.73±2.22	8.77±2.23	−0.06	0.95

*Scores of different impulsivity and Sensitivity to Punishment and Reward Questionnaires, by gender and weight status groups (mean ± s.d.). BIS, Behavioral Inhibition System, Carver and White (1994); BAS, Behavioral Activation System, Carver and White (1994); BIS-11, Barrat Impulsiveness scale 11- Total score, 1985*.

### Subjective time perception

In order to test for a potential influence of subjective time perception on inter-temporal decision-making preferences we assessed subjective perception of the length of a time interval of 2, 4, and 12 months on a visual analog scale (Zauberman et al., 2009; Cooper et al., 2013) and fitted a Power-function to the data (Cf. Kim and Zauberman, 2013). To detect potential group differences we compared the parameters α and β taken from the Power-model between subgroups. However, the analysis revealed no significant main effect for gender [α: *F*_(1, 50)_ = 3.66, *p* = 0.06; β: *F*_(1, 50)_ = 0.11, *p* = 0.75], obesity [α: *F*_(1, 50)_ = 0.02, *p* = 0.88; β: *F*_(1, 50)_ = 0.00, *p* = 0.99], or for the interaction of gender × obesity [α: *F*_(1, 48)_ = 0.09, *p* = 0.76; β: *F*_(1, 48)_ = 1.64, *p* = 0.21]. Therefore, we assume that potential differences in inter-temporal preferences between groups were independent of subjective time perception.

### Model comparisons of discount functions

Choices in the temporal discounting task were best described by a quasi-hyperbolic function (mean BIC values of the model fitted to responses in RC task of the Baseline session: exponential model: 130.1 (SEM = 4.3; median = 130.8); hyperbolic model: 111.5 (SEM = 4.5; median = 106.8); quasi-hyperbolic model: 106.5 (SEM = 4.3; median = 98.8). Consequently, all following analyses were based on the quasi-hyperbolic model.

### Group differences in discounting

To investigate differences in inter-temporal decision-making between lean and obese as well as male and female participants we conducted group analyses on the parameters of the quasi-hyperbolic model.

The mean value across the entire sample of the present bias β was 0.72 (SD = 0.26). A two-way analysis of variance showed no main effect for weight status [*F*_(1, 51)_ = 1.21; *p* = 0.28], no main effect for gender [*F*_(1, 51)_ = 0.39; *p* = 0.54], and no interaction between weight status and gender [*F*_(1, 51)_ = 1.38; *p* = 0.25].

**Figure 2:**
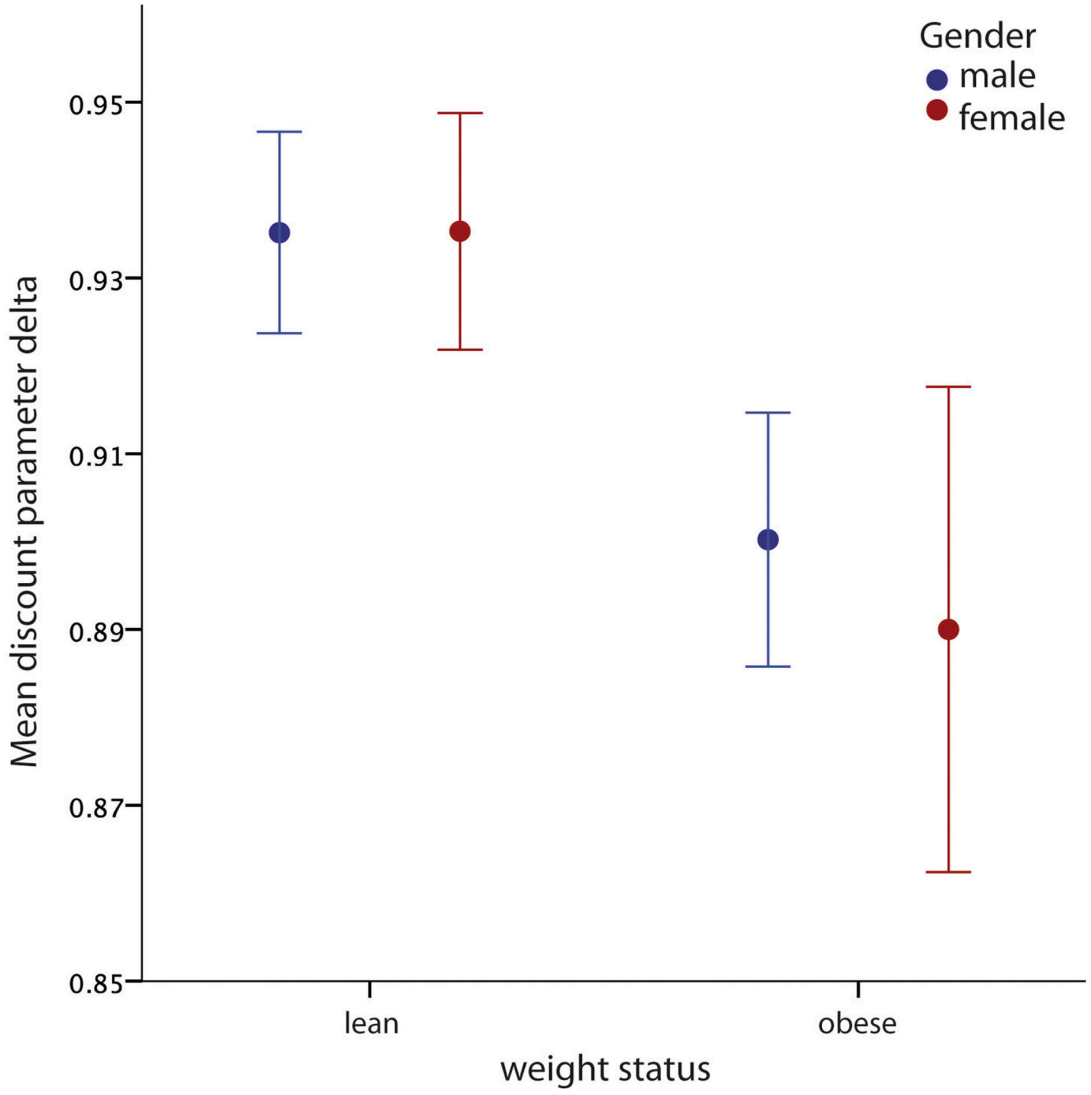
**Main effect of obesity on delay discounting parameter δ**. Obese subjects had lower values of the discounting parameter δ, independent of gender. Error bars indicate standard errors of means (SEM).

The mean value of the discount factor δ was 0.92 (SD = 0.07). A two-way analysis of variance showed a main effect for weight status group [*F*_(1, 51)_ = 5.02; *p* = 0.03], with obese participants exhibiting lower values of δ, meaning that obese participants discount future rewards at a higher rate per unit of time delay than lean (see Figure 2). If this effect were dependent on socioeconomic status one would have expected an effect in the opposite direction given that members of the obese group had on average higher incomes than members of the lean group. The analysis did not show a significant effect for gender [*F*_(1, 51)_ = 0.079; *p* = 0.78], and no significant interaction between weight status group and gender [*F*_(1, 51)_ = 0.084; *p* = 0.77].

### Relationship between discount parameters and questionnaire data

**Figure 3:**
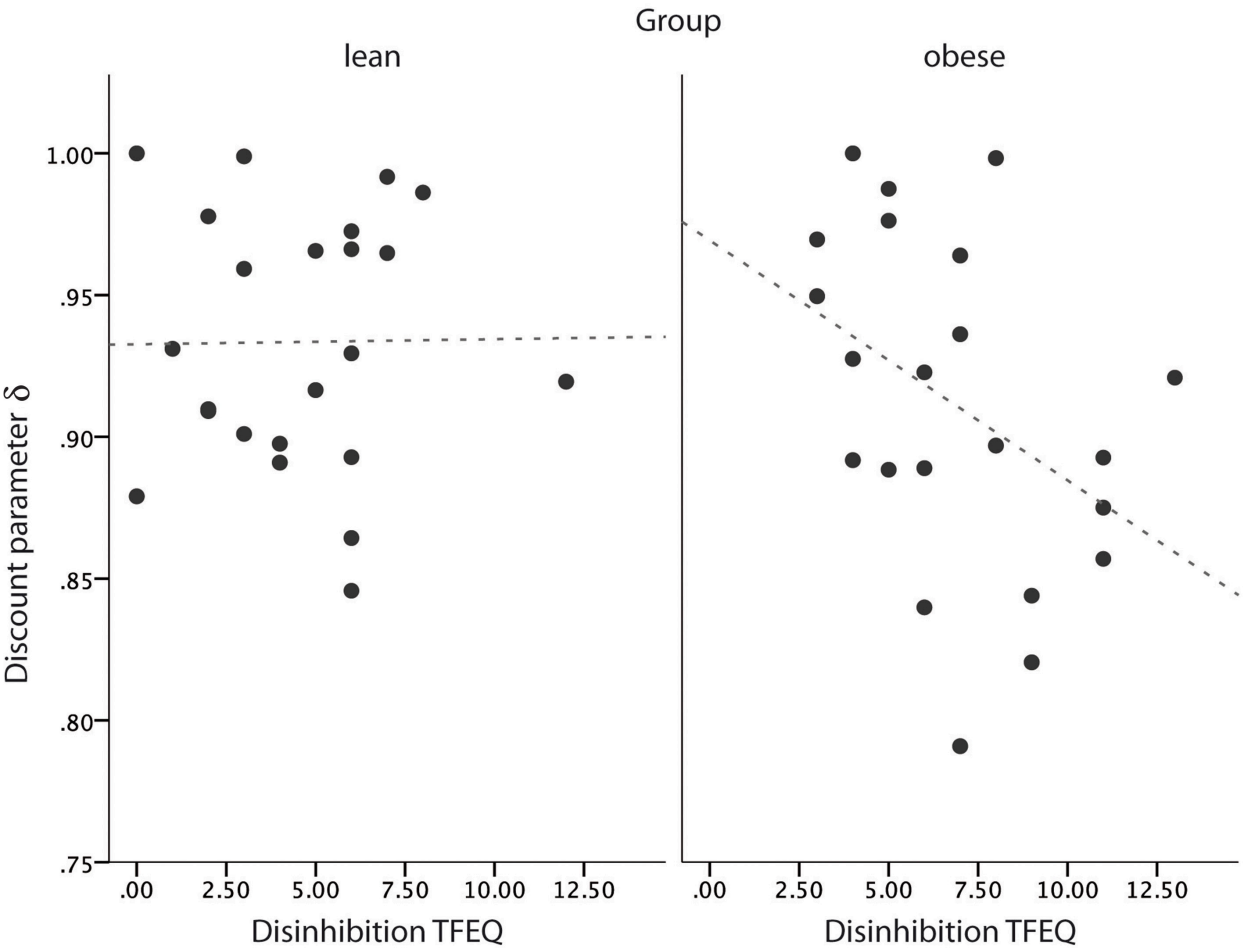
**Relationship of delay discounting parameter δ to self-reported disinhibition of eating scores from the Three-Factor Eating Questionnaire (TFEQ) in the obese group**. Dotted line equals trend line.

Given that decisions in the eating behavior context often resemble inter-temporal decision-making dilemmas, we aimed at testing if delay discounting task performance was directly related to disinhibition scores from the Three-Factor Eating Questionnaire (TFEQ). We correlated scores of the subscale disinhibition, which measures the degree of a propensity to overeat in an obesogenic environment, with δ taken from the quasi-hyperbolic model. We found that in obese in contrast to lean subjects, the discount factor δ scores were inversely correlated to disinhibition scores reflecting opportunistic eating [see Figure 3, Spearman's ρ (obese) = −0.46, *p* = 0.03; Spearman's ρ (lean) = 0.06, *p* = 0.79; two-tailed]. In other words, we found that a fast decrease of subjective value over time is directly related to a tendency to opportunistic eating. This relationship holds true for obese participants only.

Based on a strong conceptual overlap of delay discounting and self-report measurements of impulsivity/sensitivity to reward, we further hypothesized the existence of a relationship between measures of these two concepts. The present bias β was positively correlated with BAS reward scores, indicating the degree of a person's positive response to a reward—anticipated or occurring—, in the lean group [Spearman's ρ (lean) = 0.44; *p* = 0.03; two-tailed] but not in the obese group [Spearman's ρ (obese) = 0.08, *p* = 0.68; two-tailed]. Conversely, β was negatively correlated with U-P-P-S Urgency scores, measuring the strength of experienced impulses, especially under negative affect, in the obese group [Spearman's ρ (obese) = −0.43; *p* = 0.03; two-tailed], but entirely uncorrelated in the lean group [Spearman's ρ (lean) = −0.001, *p* = 0.99; two-tailed]. The discount factor δ was negatively correlated with BAS reward scores in the entire sample (Spearman's ρ = −0.46; *p* = 0.01; two-tailed). *Post-hoc* analyses showed that this effect was stronger in the obese group (Spearman's ρ (obese) = −0.54, *p* = 0.01; two-tailed) and marginally failed to reach significance in the lean group [Spearman's ρ (lean) = −0.38, *p* = 0.06; two-tailed].

Finally, across groups we found that men, in contrast to women, exhibited a positive correlation between BIS-15 motor impulsivity scores and the stochasticity parameter *s* [Spearman's ρ (men) = 0.46, *p* = 0.02; Spearman's ρ (women) = 0.15, *p* = 0.46; all two-tailed]. Men, in contrast to women, exhibited a direct relationship between a tendency to act without thinking (motor impulsivity) and a low sensitivity toward changes in reward magnitude in a delay discounting task, as described by high values of *s*.

### Reaction times and inter-temporal decision-making

We also investigated the relation between reaction times (RT) and inter-temporal decision-making. Our analysis was based on a correlational analysis of RTs and discount factors (δ parameter in the discount function), quantifying the rate of de-valuation of future rewards. Discount factors were computed separately for each of the two tasks in the priming session. RTs exhibited significant gender differences in both sessions, with women exhibiting faster RTs (for detailed statistics see Table 3). Additionally, RTs in the PRC task of the Priming session were faster by trend in the obese group [*F*_(1, 50)_ = 3.84, *p* = 0.06]. This difference was significant when considering easy trials only [*F*_(1, 50)_ = 4.97, *p* = 0.03]. There was no significant difference between RTs in the different priming conditions.

To test if the observed differences in RTs were related to discounting behavior, we correlated RTs with participants' discount factors in the RC and the PRC task of the priming session, separately for gender groups. We found that for men, independent of the weight status, RTs were positively correlated with discount factors, implying that fast responses during inter-temporal choices were associated with higher rates of discounting of future rewards [*for the RC task*: Spearman's ρ (men) = 0.44, *p* = 0.03; Spearman's ρ (women) = 0.24, *p* = 0.24; all two-tailed; *for the PRC task:* (a) neutral condition: Spearman's ρ (men) = 0.49, *p* = 0.01; Spearman's ρ (women) = 0.28, *p* = 0.17; all two-tailed; (b) social condition (Spearman's ρ (men) = 0.42, *p* = 0.05; Spearman's ρ (women) = 0.3, *p* = 0.14; all two-tailed; (c) erotic condition: (Spearman's ρ (men/women) = 0.34/0.16; all *p* > 0.05; all two-tailed; (d) food condition Spearman's ρ (men/women, food) = 0.39/0.12; all *p* > 0.05; all two-tailed].

### General stability of inter-temporal decision-making preferences

We investigated two, presumably independent, aspects of stability in inter-temporal decision-making preferences. First, we examined the general stability of inter-temporal decision-making preferences. We tested for the longitudinal stability of preferences (between sessions) and for the stochasticity of decision-making, a marker of the consistency of decisions and thus the stability of internal value representations.

To examine participants' temporal stability of decision-making preferences, we compared discount factors between sessions. We applied a Fisher-r-to-z transformation to the coefficients of correlation between discount factors in the Baseline and the Priming session. We found that correlations of discount factors between sessions were high and not significantly different between the lean and obese group [*Z* = 0.52, *p* = 0.6, two-tailed; Pearson's *r* (lean) = 0.89; Pearson's *r* (obese) = 0.92; correlations significant at the *p* < 0.01 level], and men and women [*Z* = 0.52, *p* = 0.06, two-tailed; Pearson's *r* (women) = 0.89; Pearson's *r* (men) = 0.92; correlations significant at the *p* < 0.01 level]. This suggests that inter-temporal preferences in both groups were relatively stable over time in the absence of incidental rewarding cues.

Next, we tested for differences in the degree of stochasticity of participants' behavior, as captured by parameter *s* in the quasi-hyperbolic discount function. Analysis of group differences for parameter *s*, a marker for the consistency of decisions and thus the internal stability of value representations [M (SD) = 4.24 (5.45)], revealed no significant main effect for obesity [*F*_(1, 51)_ = 0.23; *p* = 0.64] or gender [*F*_(1, 51)_ = 0.55; *p* = 0.46] and no significant interaction [*F*_(1, 51)_ = 1.32; *p* = 0.26], suggesting that the stability of internal value representations in obese and lean participants does not differ in the absence of contextual cues (see above).

In sum, analysis of the general stability of inter-temporal decision-making preferences—temporal stability and consistency of decisions—revealed no differences between lean and obese.

### Susceptibility of inter-temporal decision-making to incidental rewarding pictures—impact of priming

Next, we investigated the influence of external factors on inter-temporal decision-making to measure the stability of inter-temporal preferences with regard to changes of context. To do so, we tested if incidental rewarding stimuli (the affective pictures from different context categories) had a significant priming effect on inter-temporal decision-making. Further, we analyzed whether this priming effect was systematically related to weight status and gender.

Our analysis was based on a comparison of discount factors (parameter δ) between priming conditions, with the neutral (non-priming) condition as an intra-individual baseline measure. Separate repeated-measures ANOVAs were performed to compare potential priming effects for each of several categories of images (erotic, food, social, see Table 2) between gender and weight status groups.

First, we investigated the within-subject effect of priming in separate ANOVAs for each image category in the entire sample. The analysis revealed no significant priming effect for any of the image categories [*F*_food(1, 47)_ = 0.15, *p* = 0.7; *F*_social(1, 47)_ = 0.02, *p* = 0.88], even though the erotic priming effect revealed trend-significance [*F*_erotic(1, 48)_ = 3.24, *p* = 0.08]. There was no significant main effect on priming for weight status in any category [*F*_erotic(1, 47)_ = 2.31, *p* = 0.14; *F*_food(1, 46)_ = 0.23, *p* = 0.64; *F*_social(1, 46)_ = 0.03, *p* = 0.87] nor was there a main effect for gender [*F*_erotic(1, 47)_ = 0.03, *p* = 0.87; *F*_food(1, 46)_ = 0.79, *p* = 0.38; *F*_social(1, 46)_ = 2.45, *p* = 0.13].

**Figure 4:**
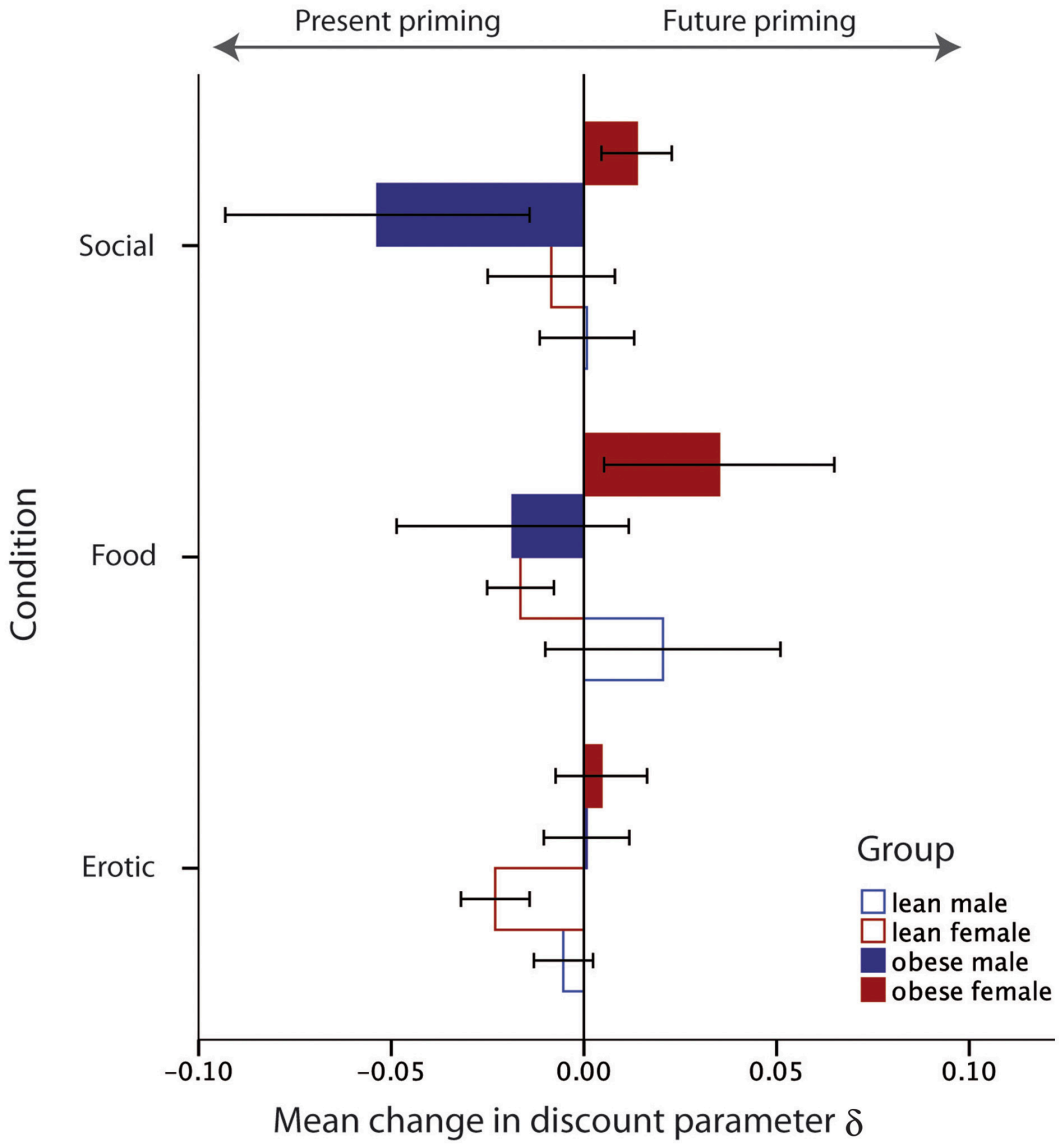
**Illustration of the interaction between gender and weight status on the priming effect for the different priming categories**. Values below zero indicate priming toward more present choices, values above zero indicate priming toward more future choices. Error bars indicate the standard errors of means (SEM).

Second, we tested for gender × obesity interactions. We found a gender × obesity effect for the food category as well as the social category [*F*_food(1, 44)_ = 4.21, *p* = 0.04; *F*_social(1, 44)_ = 4.24, *p* = 0.04], but no effect for the erotic category [*F*_erotic(1, 44)_ = 0.09, *p* = 0.76]. The interaction revealed that obese women's choices, after exposure to visual cues from the food and social category, tended to be more future-oriented while obese men's choices were more present-oriented (see Figure 4).

### Relationship between priming effect size and self-report measures of impulsivity, sensitivity to reward (SR), general intelligence, and inter-temporal preferences

We further analyzed whether the strength of subjects' susceptibility toward external rewarding cues was related to self-report measures of impulsivity (BIS-15 and U-P-P-S impulsivity questionnaire), SR (BIS/BAS scores), general intelligence (Wiener Matrizen Test), and parameters of the discount function. No significant correlation between the priming effect and self-report measures of impulsivity, SR or general intelligence was found in any category (all Pearson and rank correlations p > 0.05). However, we found a significant correlation between the priming effect of erotic images with β, the parameter capturing a “present bias” in discounting (Spearman's ρ = 0.45, *p* = 0.01). Single correlation analyses showed that the effect was strongly driven by the lean group [Spearman's ρ (lean) = 0.6, *p* = 0.01; Spearman's ρ (obese) = 0.15, *p* = 0.53] and men [Spearman's ρ (men) = 0.57, *p* = 0.01; Spearman's ρ (women) = 0.28, *p* = 0.2] and most pronounced in lean men [Spearman's ρ (lean men) = 0.83, *p* = 0.01]. This means that the lower the “present bias” in discounting, the higher the degree of susceptibility of inter-temporal choices to priming by erotic pictures, especially in lean men. However, the results have to be treated with caution given that the priming effect for erotic pictures proved only trend-significant (see above).

## Discussion

We investigated differences in inter-temporal choice between people with obesity and lean control subjects. Our results showed that obese subjects, independent of gender, devalued future rewards at higher rates than healthy controls. The groups did not differ in the stability of discounting behavior over time. Further, we showed that a higher tendency of opportunistic eating was associated with a higher reliance on immediate monetary rewards in the obese group. In addition, we found that obese subjects were more susceptible to external cues. This effect was gender-specific in its direction: food and social cues reduced discounting in obese women while they increased discounting in obese men. These findings indicate that the specific context of decision-making may play a substantial role in obesity and that this effect is not attributable to less stability in value representations in obese people.

### Higher discount rates in obese subjects

An overreliance on immediate gratification can be assumed in the context of dietary choices in obesity (Epstein et al., 2010). Interestingly, our finding that obese in contrast to lean participants also exhibited a strong preference for immediately available monetary rewards suggests obesity-associated alterations in domain-general decision-making processes. A strong preference for immediate rewards might be caused by differences in the computation of the value of the choice options. Recent studies showed structural and functional alterations of components of the valuation system in obesity (Stoeckel et al., 2008; Horstmann et al., 2011; Mueller et al., 2011; Nummenmaa et al., 2012; García-García et al., 2014). Hence, one possibility is that the common mechanism underlying the domain-general preference for immediate rewards is an altered computation of decision values in individuals with obesity. Dopamine has been shown to play an important role in cost-benefit decision-making (Treadway et al., 2012) and motivation (e.g., Hoebel et al., 2007; Kobayashi and Schultz, 2014). Human obesity and diet-induced obesity as well as a prolonged high-fat diet in rodents have been shown to produce pronounced alterations in several components of dopaminergic transmission (Cone et al., 2013; Narayanaswami et al., 2013; Sharma and Fulton, 2013; Horstmann et al., 2015b) and the modulatory endocannabinoid system (Cheer et al., 2004; Bello et al., 2012). Convergent evidence highlights alterations in dopamine D2 receptor availability, dopaminergic tone, and the efficacy of dopamine transporter (DAT). Thus, function of structures receiving dopaminergic input and playing an important role in inter-temporal choice such as ventral striatum, amygdala, and prefrontal cortex might be compromised in obesity. In the context of inter-temporal decision-making, subjective value of the options is represented in ventral striatum, medial prefrontal cortex and posterior cingulate cortex (Kable and Glimcher, 2007, 2010), while activity in anterior cingulate cortex supports the context-sensitive dynamic adjustment of preference functions via input from amygdala, posterior cingulate cortex, and hippocampus (Peters and Büchel, 2010). Recent research suggests an even more pronounced role of the basolateral amygdala as a central integrator of reward value, its history and cost parameters (Wassum and Izquierdo, 2015), which might be of particular interest in the context of inter-temporal reward-based decision-making. Altered dopaminergic input into these areas might thus produce (a) a general shift in delay discounting preferences and (b) a higher susceptibility of choice parameters to the influence of external cues. In line with this hypothesis, other studies demonstrated alterations in reward-based decision-making or impulse-control in obese populations. For example, obese people exhibited altered performance in the Iowa Gambling Task (Pignatti et al., 2006; Brogan et al., 2010; Horstmann et al., 2011; Koritzky et al., 2012), the stop signal task (Nederkoorn et al., 2006) and a Go/No-Go task (Batterink et al., 2010; Kamijo et al., 2012) when compared to their lean counterparts.

Prior studies showed that obese women discount future rewards steeper than lean women while no difference was observed for men (Weller et al., 2008). In contrast, we found comparable obesity-associated differences in discount rates in both men and women. Our study differs in important methodological aspects from the previous ones, which might have contributed to the differential findings. In their task, Weller and colleagues used hypothetical rewards with no real consequences for participants, as well as very large monetary amounts (500$ up to $50,000) and very long delays (up to 10 years). Even though the impact of the influence of reward type (real vs. hypothetical rewards) remains debated (Johnson and Bickel, 2002; Madden et al., 2003, 2004; Hinvest and Anderson, 2010), recent results showed that the use of real monetary incentives in delay discounting tasks led to shallower delay discounting functions when compared to hypothetical monetary incentives (Hinvest and Anderson, 2010). In addition, it has been shown in a delay discounting task that higher monetary amounts led to shallower discount functions, which has been termed the “magnitude effect” (Thaler, 1981; Green et al., 1999; Estle et al., 2006; Mitchell and Wilson, 2010). In our study, we used smaller monetary amounts, but participants had the chance to win one of the choices made during the experiment, creating real incentives for task performance and making our task more realistic.

Further, we carefully matched gender and weight status groups for general intelligence, age, income, and education, as these factors seem to be linked to inter-temporal decision-making preference (Green et al., 1996; Shamosh et al., 2008; Davis et al., 2010). Another possible confound that we can rule out is general differences in time perception (Takahashi, 2005; Wittmann and Paulus, 2008; Zauberman et al., 2009), as our study explicitly controlled for subjective time perception and found no differences between groups.

Having established general differences in inter-temporal choice between obese and lean people, our results further provide first evidence for a direct link between characteristics of eating behavior and inter-temporal preferences. A higher tendency of opportunistic eating was associated with a higher reliance on immediate monetary rewards in the obese group. Note, however, that the association between general inter-temporal decision-making preference and BMI cannot be fully explained by “opportunistic eating,” since correlation strength was only moderate. Other obesogenic characteristics of behavior, not covered by the “opportunistic eating” scale, are most likely additionally associated with general inter-temporal decision-making preference. These might include factors adding to an imbalance of energy uptake and energy expenditure, e.g., a lack of physical activity. In order to clarify the mechanisms by which inter-temporal decision-making preference exerts an influence on body weight, future research should thus directly address the relationships between inter-temporal decision-making preferences and the composition of diet, e.g., the variety of the diet (McCrory et al., 1999), the amount of consumed ultra processed and convenience foods (St-Onge et al., 2003; Monteiro et al., 2011), and the composition of calorie intake (Austin et al., 2011), as well as the amount of physical activity.

### Susceptibility to environmental cues

Our second major finding was an enhanced susceptibility to environmental cues in inter-temporal decisions for obese people as compared to healthy controls. Priming with rewarding images led to a differential effect in obese women compared to obese men. More specifically, after priming with visually depicted social scenes (family and happy old people) and food, obese men's choices became more present-oriented whereas obese women's choices became more future-oriented. Lean subjects did not show any significant susceptibility to the visual cues.

Priming has been shown to be effective in a number of circumstances, including inter-temporal choice (Van den Bergh et al., 2008; Zauberman et al., 2009; Murawski et al., 2012; Luo et al., 2014). It is based on the assumption that the presentation with an environmental cue biases the response to a subsequent cue, which can be entirely unrelated, due to an implicit memory effect activating association networks (Bargh, 1990; Tulving and Schacter, 1990; Chartrand and Bargh, 1996) or to the fast and automatic extraction of decision-relevant aspects of the stimuli during exposure (Bode et al., 2014).

Recent research suggests that the direction of priming effects observed in a general population depend on the perceived valence of the cues utilized. The induction of positive and negative affect by visual cues, i.e., happy and sad faces, has been shown to lead to more present-oriented choices for positive cues and more future-oriented choices for negative cues (Luo et al., 2014). Given the opposing direction in obese men and women, it is reasonable to assume that differential valuation and processing of the presented cues might moderate the effect.

Obese men, on the one hand, were biased toward immediate monetary rewards when presented with images of social scenes and food. This is in line with our expectations, as food items in particular have a high rewarding value in obesity (Rothemund et al., 2007; Wang et al., 2009; Nijs et al., 2010) and strong rewarding cues have been shown to bias a normal population toward immediate rewards (Van den Bergh et al., 2008; Murawski et al., 2012; Cooper et al., 2013; Van der Wal et al., 2013).

The future priming effect in obese women on other hand might be explained by a differential perception of the cues utilized. We hypothesize that obese women might have perceived the presented social and food cues as more negative than obese men due to differences in the implicit attitudes toward them. Note that while such attitude biases were not expressed in the *explicit* valence ratings for the presented pictures that did not differ between gender groups, the persistence of *implicit* attitude differences has been described before.

Obese women have been shown to exhibit more negative implicit associations with food, especially high-fat food items (Roefs and Jansen, 2002). Furthermore, it has been shown that women in general had stronger inhibitory DLPFC activation in response to food stimuli, leading to less *ad libitum* food intake (Cornier et al., 2010).

This might apply to social cues as well. Research shows that obese women in particular seem to suffer from the stigmatization of obesity (Puhl and Heuer, 2009), especially in relationship and social settings (Chen and Brown, 2005; Sheets and Ajmere, 2005). Given that the presented cues mirror typical social and relationship settings, obese women might have evaluated the cues implicitly as more negative than their male counterparts.

Luo et al. (2014) suggested a mechanism by which negative affect could change decision-making preferences relying on research showing that negative affect led to more goal-directed decision strategies (Forgas, 1991) and systematic processing (Loewenstein and Lerner, 2003). They used priming with sad faces to induce negative affect and attributed priming toward more future-oriented decisions to an ‘inhibition spillover effect’. This theory states that inhibition in one domain, e.g., inhibiting the emotional response to an affective visual cue, leads to an inhibition in other domains, e.g., motor responses. This effect has been hypothesized to be mediated by the right inferior frontal cortex (rIFC), which is involved in domain-general inhibitory processes (Berkman et al., 2009).

In the same way, the “inhibition spillover” effect could explain the effect of environmental cues, implicitly valued negatively by obese women, on inter-temporal choice in our study. If some stimuli were perceived implicitly as negative and not as rewarding, this may have necessitated an inhibition of the triggered emotional response, and an “inhibition spillover” might have reversed the expected decision pattern, priming obese women toward more future-oriented choices.

Our results reveal a higher degree of susceptibility toward environmental cues in obese. In obese men, in accordance with findings in general populations, priming led to more immediate choices. In contrast, obese women were primed toward more future choices. For obese men, our results indicate that rewarding environmental cues might be detrimental in that they lead to an overreliance on immediate rewards in today's enhanced food environment aiming at inducing dietary short-sightedness (Hill and Peters, 1998). For obese women, in contrast, our results reveal a decision-making pattern that appears to be rather protective in such an environment. However, in (other) addictive disorders the degree of consumption of the drug of choice is not directly linked to its implicit evaluation (Larsen et al., 2012), which is often negative (Wiers et al., 2002; Roefs et al., 2011). Transferred to the context of obesity, this might signify that the degree of consumption of food is not linked to its implicit evaluation. Thus, further research is required to establish a causal link between such a priming effect and consumption of food in the natural environment.

### Conclusions

Our findings contribute to a growing body of literature that challenges the notion that obesity is purely a medical condition (Rippe and McInnis, 2001) but instead point to a strong cognitive dimension, which can be shaped by environmental factors. Subjects with obesity exhibit impairments in different cognitive domains such as executive function, working memory (Fitzpatrick et al., 2013), learning tasks (Coppin et al., 2014) and behavioral control (Horstmann et al., 2015a), possibly mediated by non-linear changes within the central balance of phasic and tonic dopaminergic signaling (Wang et al., 2001; de Weijer et al., 2011; Dunn et al., 2012; Guo et al., 2014; Horstmann et al., 2015a). Importantly, this indicates that treatment options focusing on dietary changes and induction of physical activity only might fall far too short. Instead, treatment might need to address executive functioning (Hall et al., 2013; McClure and Bickel, 2014), impulse control, and working memory training (Shamosh and Gray, 2008; Shamosh et al., 2008; Bickel et al., 2011) in order to shield decisions from environmental influences. Further, pharmacological treatment options that target alterations that might underlie both detrimental eating behavior and cognitive impairments might prove successful. Possible targets are dopaminergic transmission (e.g., Horstmann et al., 2015a), μ-opioid receptor transmission (Cambridge et al., 2013; Laurent et al., 2014; Sanchez-Roige et al., 2014) as well as the endocannabinoid receptor system (Boomhower et al., 2013; Watkins and Kim, 2015). These systems are most likely to mutually affect reward-based decision-making in goal-directed behavior (see e.g., Labouèbe et al., 2013).

### Conflict of interest statement

The authors declare that the research was conducted in the absence of any commercial or financial relationships that could be construed as a potential conflict of interest.
