# Supplementary material for: Incidental rewarding cues influence economic decisions in people with obesity
Source: Front Behav Neurosci. 2015 Oct 15;9:278. doi: 10.3389/fnbeh.2015.00278 (PMC4606016; doi:10.3389/fnbeh.2015.00278)
Supplement: Supplementary file 1 [file DataSheet1.DOCX]

**Appendix 1- IAPS Picture numbers used in the** Rating task (pictures selected for the use in the priming task are bold):

**Erotic condition:** **4599**, 4607, 4623, 4625, **4645**, **4650**, 4653, 4651, 4660, **4676**, 4677, **4693,**  **4695**, 4698

**Food condition:** 7282, 7291, 7330, 7340, **7351**, **7400**, **7405**, 7410, 7430, 7451, 7460, **7470**, **7480**, **7481**, 7482, 7487

**Social condition:** 2057, **2152**, 2154, 2155, **2165**, 2170, 2311, 2360, **2370**, 2398, **2495**, **2500**, **2510**, 2530, 2550, 4622, 4626, 7530, 8500, 8502, 8503, 8510, 8531
